# Time-course of neuropsychological functioning in aneurysmal subarachnoid hemorrhage and its association with vasospasm

**DOI:** 10.3389/fneur.2025.1711507

**Published:** 2026-01-06

**Authors:** Giorgia Abete-Fornara, Claudia Fanizzi, Elena Scagliotti, Giorgio Fiore, Valeria Conte, Fabrizio Ortolano, Tommaso Zoerle, Marco Locatelli, Giulio Andrea Bertani

**Affiliations:** 1 Unit of Neurosurgery, Fondazione IRCCS Ca' Granda Ospedale Maggiore Policlinico of Milan, Milan, Italy; 2 Neuroscience Intensive Care Unit, Department of Anesthesia and Critical Care, Fondazione IRCCS Ca' Granda Ospedale Maggiore Policlinico of Milan, Milan, Italy; 3 Department of Medical-Surgical Physiopathology and Transplantation, University of Milan, Milan, Italy

**Keywords:** aneurysmal subarachnoid haemorrhage, cognitive impairment, daily independence, follow-up, vasospasm

## Abstract

**Background:**

Aneurysmal subarachnoid hemorrhages (aSAH) and related vasospasm often cause several neurological and cognitive impairments in survivors. The long-term impact of aSAH and vasospasm on cognition remains a topic of debate. This study aims to describe cognitive functioning, focusing on the acute phases after bleeding and for 18 months, and to investigate the immediate and long-term effects of vasospasm.

**Methods:**

Seventy adult patients were prospectively recruited and tested at different time points: within 48/72 h after bleeding (T1); between 7 and 10 days after bleeding (T2); and five long-term follow-ups from 1 (T3) to 18 months (T7). An extensive neuropsychological evaluation was administered, including the level of daily functional independence.

**Results:**

At T1, all tests showed high percentages of impairments (ranging from 38 to 100%), in particular for visual and verbal long-term memory, constructional praxis, abstract reasoning, and functional independence. Many tasks have gradually improved since T2, except for executive functions and visual memory, which show a slower recovery. A severe diffuse impact of vasospasm emerged at T2, but a linear, gradual recovery emerged since T3 for almost all the investigated functions. At the last follow-ups, several tests showed no significant differences between patients with and without vasospasm.

**Conclusion:**

Despite severe diffuse impact of bleeding and vasospasm in the acute stages, a low prevalence of cognitive and functional impairments in the chronic phase emerges. Our data may help clinicians to better understand the cognitive and autonomy trajectories of recovery over time and to tailor eventual rehabilitation programs.

## 1 Introduction

Intracranial aneurysmal subarachnoid hemorrhage (aSAH) is a severe kind of stroke related to aneurysm rupture associated with high rates of morbidity and mortality due to focal and diffuse brain damage and augmented intracranial pressure. Several studies reported mortality percentages of 15–35% in the first 48 h, with rates of 50% after 1 month, despite recent improvements in clinical management (1–3). Such a poor prognosis also depends on subsequent mechanisms, such as vasospasm and the related delayed cerebral ischemia (DCI). The former is a very common complication consisting of blood vessel contraction, with nearly 70% of aSAH patients developing it (4), usually starting 3 days after the first bleeding, and which significantly worsens clinical outcomes and prognosis (5). DCI, also known as clinical or symptomatic vasospasm, is a complex ischemic event occurring in 17 to 40% of patients with aSAH and results from arterial shrinkage. As a result, survivors often experience severe or moderate neurological and/or cognitive impairments that interfere with everyday life functioning and autonomy (6–8). Among the most frequently reported cognitive impairments, studies describe attention and executive function deficits, lasting also for 1–3 years after aSAH (6, 7, 9). Verbal memory is another function highly impaired in both subacute and chronic stages, with significant percentages of deficits even after several years of bleeding (10–12). Moreover, memory is reported to be the slowest function to recover compared to other cognitive and neurological abilities (10). Impaired cognition is also responsible for difficulties in returning to work and in daily autonomy, as measured by the Activities of Daily Living Scale (ADL) and Instrumental Activities of Daily Living Scale (IADL), which have been reported to indicate general long-term impaired autonomy, probably underestimated (11–13).

Neural mechanisms underlying cognitive impairments and their correlations with clinical and radiological aspects are still an open debate: A systematic review concerning cognition in SAH (13) reported inconsistent findings concerning possible correlations among neuropsychological functions and clinical parameters such as edema, site or side of bleeding, or neurological status at admission. The only stable parameter is represented by the treatment method of the aneurysm, with better cognitive outcomes in patients treated with coil than clipping (3, 14), and left-sided bleeding, associated with worse language outcomes (15, 16).

In general, recent studies suggest that acute clinical factors concerning a SAH, such as its severity or the thickness of the subarachnoid blood, even if involved in cognitive outcome, are not its primary determinants, as they account for only low percentages of the variability (13, 17, 18). Rather, subsequent mechanisms such as vasospasm, intracranial pressure, or DCI are hypothesized to primarily contribute to neuropsychological impairment, even if opposite findings have also been described (10).

Furthermore, data available in the literature differ in the timing of the neuropsychological evaluation, with a greater number of studies dealing with long-term cognitive outcome than with acute or subacute stages.

In this view, the present study aims to prospectively investigate the time course of neuropsychological functioning during the first 18 months after aneurysmal SAH in adult patients, with a specific interest in the first hours/days after bleeding, and to investigate the immediate and long-term effects of vasospasm.

## 2 Materials and methods

### 2.1 Patient recruitment

Patients with aSAH were prospectively recruited from the intensive care unit (ICU) and Neurosurgery Unit at Fondazione IRCCS Ospedale Maggiore Policlinico of Milan from November 2011 to November 2016. Patients were considered eligible according to the following criteria: a) having more than 18 years of age; b) being an aSAH confirmed at the angiography or angio-tomography (angio-TC), independently from site or side; c) being able to undergo a neuropsychological evaluation at least at one of the first two time-points scheduled (see below); d) being able to perform a magnetic resonance imaging (MRI) and angio-MRI during the first 48 h from the event; and e) in the case of foreign patients, proving adequate Italian language skills to understand medical information and undergo cognitive testing.

Written informed consent was signed by patients if conscious and able to understand; otherwise, a significant relative signed on his/her behalf. In consideration of inclusion criterion c), in the case of a patient unconscious at T1 whose caregiver signed the written consent to medical and cognitive testing, his or her level of consciousness and cooperation and acceptance to undergo cognitive testing were checked at T2 before eventual enrollment and consequent neuropsychological testing.

Our local ethical committee approved this project, which is performed in line with the principles of the Declaration of Helsinki and approved by the Institutional Review Board of Foundation IRCCS Ca′ Granda Ospedale Maggiore Policlinico for studies involving humans.

### 2.2 Clinical and neuropsychological evaluations

Patients underwent a complete neuropsychological evaluation at several time points: within 48/72 h after bleeding (acute phase, T1); between 7 and 10 days after bleeding (sub-acute phase, T2); five long-term follow-ups after 1 (T3), 3 (T4), 6 (T5), 12 (T6), and 18 months (T7). The neuropsychological battery included standardized and validated tests to assess language, short- and long-term memory for visual and verbal stimuli, constructional, ideomotor, and orofacial praxis, attention, and executive functions. Sustained attention is also qualitatively assessed through the observation of patients’ behavior during the exam. Parallel versions were used when available to avoid the learning effect among the evaluations. Raw scores of each test were corrected for age, educational level, and gender based on Italian normative data in order to calculate the adjusted scores; equivalent scores were also calculated: an equivalent score of 0 or 1 was considered impaired, according to the Italian neuropsychological criteria. In addition, functional impairments in everyday life activities were assessed through the Activities of Daily Living (ADL) and Instrumental ADL (IADL) scales. The entire evaluation took approximately 1 h. No patients underwent cognitive rehabilitation during the follow-up period.

In our sample, we checked for eventual unilateral spatial neglect (USN) through qualitative (not looking towards left or right, failing to read or detect spatial stimuli in the tests, etc.) and quantitative (the “Position Preference” score at the Raven test) indexes, as this condition could be a potential bias in some tests. Coherently with the literature, no patients showed USN symptoms. For the complete list of the tests and questionnaires used, see Table 1.

**Table 1:** Complete list of neuropsychological tests and questionnaires administered, divided into respective cognitive domains.

Cognitive domain	Test	Function tested
Memory	Digit span forward (26)	Short-term verbal memory
	Corsi span (26)	Short-term visuo-spatial memory
	Rey/Taylor complex figure-recall (27, 28)	Long-term visuo-spatial memory
	Rey 15-word list (RAVLT) immediate and long-term recall (29)	Auditory learning/long-term verbal memory
Executive functions	Digit span backward (26)	Verbal working memory
	Stroop test (30)	Cognitive inhibition and flexibility
	Weigl test (31)	Abstract and categorical reasoning
	Raven colored progressive matrices (32)	Non-verbal logical reasoning
	Clock drawing test (33)	Spatial planning, semantic memory
Language	Phonemic fluencies (34)	Verbal fluency through phonemic cue
	Semantic fluencies (35)	Verbal fluency through semantic cue
	Object naming (36)	Language production/semantic memory
	Token test (37)	Language comprehension
Attention	Attentive matrices (31)	Visuo-spatial selective attention
Praxis	Rey/Taylor complex figure (27, 28)	Constructional praxis
	Action imitation (38)	Ideomotor praxis
	Movement imitation (39)	Oro-facial praxis
Daily autonomy	Activities of Daily Living (ADL) Scale (40)	Level of independence
	Instrumental Activities of Daily Living (IADL) Scale (41)	Level of independence in instrumental activities

Concerning clinical evaluations, patients were evaluated according to the World Federation of Neurological Surgeons score (WFNS). Hemorrhagic severity was evaluated through the Fisher scale calculated at the first cerebral tomography (CT).

Aneurysms were excluded from blood flow through endovascular (coil) or surgical (clip) techniques within the first 24 h. When hydrocephalus was reported, an EVD was placed through Kocher’s point. A 3-tesla MRI and angio-MRI were performed within 48 h (T1) and 7–10 days after (T2) the aSAH, respectively. Daily monitoring of arterial blood flow was performed through transcranial eco-doppler (TCD).

### 2.3 Statistical analysis

Analyses were performed using IBM SPSS version 26 and JASP version 0.18.3. Descriptive statistics were calculated to describe the cognitive performance of the whole sample over time. T-tests for independent samples were run to study the effects of vasospasm on cognition at T2; we considered T2 the most appropriate time point to investigate the impact of vasospasm on cognition while occurring, a period and effect rarely addressed before.

In those cases, when a patient did not undergo a test due to his or her severe clinical condition (missing values); we considered the performance as obtaining an adjusted score of “0.0” (the so-called “ground score”) and consequently an equivalent score of “0,” in representation of the cognitive impairment preventing the patient from being tested.

Chi-square tests analyzed the differences between patients with or without vasospasm in several clinical variables. Mann–Whitney tests were used for ADL and IADL scales due to their non-normal distributions. Specific corrections for multiple comparisons were applied.

To explore the effects of vasospasm over time, two-way mixed repeated measures ANOVAs with Bonferroni correction for multiple comparisons were run to study the direct and interactive effects of time (within-subjects factor) and vasospasm (between-subjects factor) on the adjusted scores of the cognitive tests at different time points. Results were considered significant with a *p-*value of < 0.05.

## 3 Results

### 3.1 Patients cohort

Seventy adult patients were enrolled, with a mean age of 54.56 years (± 11.88) and a mean educational level of 11.10 years (± 3.93), 45 (64.3%) of whom were females. Sixty-six patients were Italian; the remaining four demonstrated good to excellent Italian language abilities. In 61 patients (87.1%), the hemorrhage occurred in the anterior circulation, and in almost half of the samples (33, 47.1%), it involved the anterior communicating artery. Fifty-one subjects developed no hydrocephalus. Almost all patients (65, 92.8%) received a coiling treatment, coherently with current trends in neurosurgery, where minimally invasive techniques are increasingly employed to optimize patients’ neurological and cognitive outcomes. Surgical clipping has been preferred for only five patients, due to aneurysm morphology and location or to intracranial hypertension. The complete list of socio-demographical and clinical data is reported in Table 2.

**Table 2:** Complete list of socio-demographical and clinical data for the sample.

Variable	Population		
	Whole sample	Vasospasm_yes	Vasospasm_no
Age (years): mean (SD), range	54.56 (11.88), 26–79	53.83 (10.36), 31–79	55.29 (13.34), 26–79
Education (years): mean (SD), range	11.10 (3.93), 3–18	10.97 (4.02), 3–17	11.23 (3.89), 3–18
Gender, *N* (%)			
Females:	45 (64.3%)	23 (65.7%)	22 (62.8%)
Males:	25 (35.7%)	12 (34.3%)	13 (37.1%)
WFNS, *N* (%)			
1:	26 (37.1%)	9 (25.7%)	17 (48,6%)
2:	17 (24.3%)	8 (22.9%)	9 (25.7%)
3:	11 (15.7%)	6 (17.1%)	5 (14.3%)
4:	12 (17.1%)	8 (22.9%)	4 (11.4%)
5:	4 (5.7%)	4 (11.4%)	0 (0%)
Fisher scale, *N* (%)			
1:	12 (17.1%)	5 (14.3%)	7 (20.0%)
2:	10 (14.3%)	5 (14.3%)	5 (14.35)
3:	23 (32.9%)	12 (34.3%)	11 (31.4%)
4:	25 (35.7%)	13 (37.1%)	12 (34.3%)
Circulus, *N* (%)			
Anterior:	61 (87.1%)	33 (94.3%)	28 (80.0%)
Posterior:	9 (12.9%)	2 (5.7%)	7 (20.0%)
Site of bleeding, *N* (%)*			
Anterior Communicating artery:	33 (47.1%)	20 (57.1%)	13 (37.1%)
ICA:	11 (15.7%)	3 (8.6%)	8 (22.9%)
Posterior Communicating artery:	10 (14.3%)	2 (5.7%)	8 (22.9%)
MCA:	10 (14.3%)	8 (22.9%)	2 (5.7%)
PICA:	3 (4.3%)	0 (0%)	3 (8.6%)
Basilar artery:	2 (2.9%)	2 (5.7%)	2 (5.7%)
PCA:	1 (1.4%)	0 (0%)	1 (2.9%)
Surgical treatment, *N* (%)			
Coil:	65 (92.8%)	32 (91.4%)	33 (94.3%)
Clip:	5 (7.1%)	3 (8.6%)	2 (5.7%)
Hydrocephalus, *N* (%)			
Yes	19 (27.1%)	12 (34.3%)	7 (20%)
No	51 (72.9%)	23 (65.7%)	28 (80%)

SD, standard deviation; WFNS: World Federation of Neurological Surgeons Score Scale; ICA, internal carotid artery; MCA, middle cerebral artery; PICA, posterior cerebellar inferior artery; PCA, posterior cerebral artery.

*Only this variable resulted significantly different between the groups with and without vasospasm (*p* = 0.009).

Radiological vasospasm at aMRI was detected in 35 patients (50%), whose characteristics are described in Table 2, and occurred in the anterior communicating artery for most of the sample (20 patients, 57.1%). No significant differences emerged between patients with and without vasospasm for age (*p =* 0.78), education (*p* = 0.85), or sex (*p* = 0.80), while a significant difference (*p* = 0.009) was found for sites of bleeding, independent of the hemorrhage severity as calculated with Fisher’s score. In particular, as shown in Table 2, vasospasm was more common in the anterior communicating and middle cerebral arteries (*X*^2^_(6)_ = 16.96). Twelve patients (17.1%) developed transient or permanent neurological impairment due to vasospasm assessed radiologically. The association between clinical vasospasm and neuropsychological assessment was not performed because of the unbalanced distribution of the sample.

As Table 3 reports, not all patients completed the neuropsychological evaluation at all seven time points. In the acute and sub-acute phases, a few patients did not manage to sustain the cognitive effort required to be evaluated at one of the two time points. In a few cases, patients were lost at follow-ups due to the following reasons: patient unwillingness to undergo the exam, patient inability to reach the hospital for clinical or personal reasons, or the inability of contacting him/her.

**Table 3:** Mean scores and percentages of impairments of the neuropsychological tests administered in different time points for the whole sample.

Test	Time	*N*	Mean scores (SD)	Impairments *N* (%)
Digit span forward	T_1_	60	4.71 (0.9)	22 (36.7%)
	T_2_	57	5.01 (1.0)	13 (22.8%)
	T_3_	50	5.40 (0.8)	5 (10%)
	T_4_	63	5.31 (0.8)	7 (11.1%)
	T_5_	59	5.42 (0.8)	5 (8.5%)
	T_6_	48	5.53 (0.7)	4 (8.4%)
	T_7_	35	5.57 (0.8)	2 (5.7%)
Corsi span	T_1_	57	3.61 (0.7)	41 (71.9%)
	T_2_	58	3.63 (0.9)	42 (72.4%)
	T_3_	50	4.29 (0.9)	17 (34%)
	T_4_	63	4.29 (0.9)	25 (39.7%)
	T_5_	59	4.36 (1.1)	19 (32.2%)
	T_6_	48	4.54 (0.7)	14 (29.2%)
	T_7_	35	4.59 (0.8)	10 (28.6%)
RAVLT: immediate recall	T_1_	52	10.88 (10.36)	41 (78.8%)
	T_2_	57	27.49 (12.5)	36 (63.2%)
	T_3_	49	33.76 (8.4)	21 (42.9%)
	T_4_	63	34.94 (8.7)	25 (39.7%)
	T_5_	59	38.38 (8.8)	19 (32.2%)
	T_6_	48	39.08 (8.3)	10 (20.8%)
	T_7_	32	42.56 (11.5)	6 (17.1%)
RAVLT: delayed recall	T_1_	8	3.2 (2.36)	8 (100%)
	T_2_	54	4.28 (3.4)	35 (64.8%)
	T_3_	49	6.6 (2.6)	16 (32.7%)
	T_4_	63	6.69 (3.2)	21 (33.3%)
	T_5_	59	7.51 (3.1)	14 (23.7%)
	T_6_	48	7.28 (3.3)	10 (20.9%)
	T_7_	32	8.02 (3.5)	6 (17.1%)
Complex figure: recall	T_1_	51	5.82 (5.51)	40 (78.4%)
	T_2_	58	8.5 (5.6)	39 (67.2%)
	T_3_	49	11.76 (5.7)	23 (46.9%)
	T_4_	63	13.36 (6.4)	22 (34.9%)
	T_5_	57	13.44 (6.5)	21 (36.8%)
	T_6_	47	14.09 (5.8)	14 (29.8%)
	T_7_	35	13.84 (7.0)	14 (40%)
Attentional matrices	T_1_	54	34.80 (13.2)	29 (53.7%)
	T_2_	58	36.40 (12.6)	25 (43.1%)
	T_3_	50	45.29 (10.0)	4 (8%)
	T_4_	63	46.68 (9.6)	7 (11.1%)
	T_5_	59	48.13 (8.1)	5 (8.5%)
	T_6_	48	49.98 (7.4)	2 (4.2%)
	T_7_	34	51.64 (7.1)	1 (2.9%)
Object naming test	T_1_	58	41.19 (7.5)	27 (46.6%)
	T_2_	58	42.18 (7.4)	22 (37.9%)
	T_3_	50	44.73 (2.4)	8 (16%)
	T_4_	63	44.87 (3.0)	9 (14.3%)
	T_5_	59	45.64 (2.2)	8 (13.6%)
	T_6_	46	46.15 (1.8)	5 (10.4%)
	T_7_	34	46.40 (1.6)	3 (8.8%)
Phonemic fluency	T_1_	61	18.74 (9.8)	42 (68.9%)
	T_2_	58	20.98 (12.1)	33 (56.9%)
	T_3_	50	26.32 (10.2)	18 (36%)
	T_4_	62	26.95 (10.8)	21 (33.9%)
	T_5_	59	29.32 (10.2)	14 (23.7%)
	T_6_	48	31.17 (10.5)	8 (16.7%)
	T_7_	35	31.00 (11.0)	4 (11.4%)
Token test	T_1_	55	29.07 (4.5)	22 (40%)
	T_2_	58	29.57 (5.1)	12 (20.7%)
	T_3_	49	31.72 (2.3)	1 (2%)
	T_4_	61	32.04 (3.2)	6 (9.7%)
	T_5_	57	32.79 (2.2)	2 (3.5%)
	T_6_	46	33.68 (1.8)	1 (2.1%)
	T_7_	33	34.16 (1.7)	0 (0%)
Complex figure: copy	T_1_	55	21.69 (10.7)	39 (70.9%)
	T_2_	58	29.57 (5.1)	28 (49.1%)
	T_3_	50	30.69 (5.9)	12 (24%)
	T_4_	63	31.32 (5.8)	13 (20.6%)
	T_5_	59	31.29 (6.1)	15 (25.4%)
	T_6_	47	32.54 (4.5)	10 (20.8%)
	T_7_	35	32.35 (4.7)	5 (14.3%)
Action imitation	T_1_	57	62.68 (6.1)	31 (54.4%)
	T_2_	58	62.93 (7.1)	33 (56.9%)
	T_3_	49	66.96 (3.8)	20 (40.8%)
	T_4_	62	66.89 (4.1)	21 (33.9%)
	T_5_	58	67.43 (3.8)	19 (32.8%)
	T_6_	48	68.67 (3.2)	4 (8.3%)
	T_7_	34	69.18 (3.4)	2 (5.9%)
Facial movement imitation	T_1_	57	18.63 (1.9)	22 (38.6%)
	T_2_	58	18.69 (1.7)	21 (36.2%)
	T_3_	49	19.33 (0.9)	10 (20.4%)
	T_4_	63	19.29 (1.0)	12 (19.4%)
	T_5_	58	19.52 (0.8)	7 (12.1%)
	T_6_	48	19.62 (0.7)	6 (12.5%)
	T_7_	34	19.56 (0.8)	5 (14.7%)
Semantic fluency	T_1_	61	26.33 (11.9)	34 (55.7%)
	T_2_	58	27.67 (11.7)	24 (41.4%)
	T_3_	50	33.54 (7.8)	12 (24%)
	T_4_	63	34.70 (7.4)	14 (22.2%)
	T_5_	59	36.53 (7.5)	10 (16.9%)
	T_6_	48	38.57 (6.4)	5 (10.4%)
	T_7_	35	39.19 (7.4)	4 (25.7%)
Digit span backward	T_1_	60	3.24 (0.9)	31 (51.7%)
	T_2_	57	3.25 (0.9)	27 (47.4%)
	T_3_	50	3.75 (0.7)	16 (32%)
	T_4_	63	3.97 (0.9)	12 (19%)
	T_5_	59	4.05 (0.8)	11 (18.6%)
	T_6_	48	4.17 (0.9)	9 (18.8%)
	T_7_	35	4.50 (0.8)	3 (8.6%)
Stroop test-time	T_1_	53	49.72 (50.11)	31 (50.8%)
	T_2_	55	52.78 (69.0)	27 (46.6%)
	T_3_	50	24.53 (11.4)	15 (30%)
	T_4_	61	26.03 (23.5)	13 (20.7%)
	T_5_	58	23.15 (17.9)	7 (11.9%)
	T_6_	47	20.11 (10.4)	4 (8.3%)
	T_7_	34	18.33 (10.1)	2 (5.7%)
Stroop test-errors	T_1_	53	4.44 (4.7)	25 (41%)
	T_2_	55	3.47 (5.2)	20 (34.5%)
	T_3_	50	1.22 (1.6)	7 (14%)
	T_4_	61	1.27 (1.9)	8 (12.7%)
	T_5_	58	0.86 (1.5)	6 (10.25)
	T_6_	47	0.66 (1.1)	3 (6.3%)
	T_7_	34	0.27 (0.6)	0 (0%)
Raven coloured progressive matrices	T_1_	53	27.36 (5.7)	10 (18.9%)
	T_2_	57	27.69 (5.9)	12 (21.1%)
	T_3_	50	29.77 (5.2)	7 (14%)
	T_4_	63	29.78 (4.7)	5 (7.9%)
	T_5_	58	30.77 (4.2)	3 (5.2%)
	T_6_	48	31.67 (3.4)	0 (0%)
	T_7_	35	31.59 (3.4)	1 (2.9%)
Weigl test	T_1_	54	7.15 (3.0)	45 (83.3%)
	T_2_	58	8.13 (3.3)	40 (69%)
	T_3_	50	10.16 (2.7)	22 (44%)
	T_4_	62	10.32 (2.6)	21 (33.9%)
	T_5_	58	10.81 (2.1)	17 (29.3%)
	T_6_	48	11.28 (2.1)	10 (20.8%)
	T_7_	34	11.44 (1.9)	7 (20.6%)
Clock drawing test	T_1_	61	5.83 (3.6)	33 (54.1%)
	T_2_	58	6.24 (3.7)	27 (46.5%)
	T_3_	50	8.23 (2.6)	12 (24%)
	T_4_	62	8.54 (2.6)	12 (19.1%)
	T_5_	57	8.83 (2.3)	6 (10.2%)
	T_6_	44	8.89 (2.0)	7 (14,6%)
	T_7_	30	9.45 (1.3)	2 (5.7%)
ADL	T_1_	61	1/6	
	T_2_	58	4/6	
	T_3_	50	6/6	
	T_4_	63	6/6	
	T_5_	59	6/6	
	T_6_	48	6/6	
	T_7_	35	6/6	
IADL	T_1_	61	0/8	
	T_2_	58	2/8	
	T_3_	50	6/8	
	T_4_	63	8/8	
	T_5_	59	8/8	
	T_6_	48	8/8	
	T_7_	35	8/8	

SD, standard deviation. SD is calculated only for continuous scores; thus, for ADL and IADL, being ordinal scales, SD is not provided; the median score is reported instead, together with the maximum scores for each scale.

Noteworthy, patients with a WFNS score of 4 or 5 managed to be tested at T1 or T2, obtaining also normal scores, despite their critical clinical conditions.

### 3.2 Statistical results

Concerning neuropsychological functioning over time, mean scores for each test and relative percentages of impairment for each time point are reported in Table 3. Data show that memory is highly impaired at T1 in all the sub-domains tested, with percentages of impairment ranging from around 37 to 100%. In particular, RAVLT-immediate and delayed are the tests impaired the most; the latter even reports impaired functioning in the whole sample, with only eight patients who managed to undergo it. All functions recover already in the sub-acute phase (T2), except for the Corsi span, which starts to improve 1 month after the hemorrhage and shows a gradual improvement over time. The only exception is the Figure Recall test, which maintains 40% impairment at T7.

Visual attention is impaired in around half (53.7%) of the sample at T1, while it reaches a very low percentage of impairment (3%) at T7.

Concerning language, at T1 notable percentages of impairment emerge, ranging from 40 to 68.9%; however, it shows a good recovery in both production and comprehension functions over time: at T2 all tests improve, especially the Token test, which has suffered impaired performances, from 40 to 20.7%, and obtains a complete recovery (0%) at T7. The highest impairment at T7 concerns semantic fluency (25.7%). The phonemic fluency is the most impaired test at T1 and shows the slowest recovery over time.

Furthermore, the praxis domains showed general improvement over time, with the figure copy test showing the highest percentage of impairment in the acute (70.9%) and sub-acute (49%) phases.

As far as executive functions are concerned, at T1, impairments range from 20 (Raven Matrices) to 83% (Weigl test), resulting in the most heterogeneous domain in the acute phase, with a mean general impairment of approximately 50%. Only slight recoveries appear at T2, different from the other domains, but an evident improvement emerges during the follow-ups, with significantly lower percentages of impairments at T7, for example, the stroop error score, which reports the absence of impaired performances. The Weigl test shows the highest impairment in this domain at the last follow-up (approximately 21%).

Concerning daily autonomy, a severe reduction is necessarily present at T1 in both ADL and IADL scales; interestingly, whereas ADL improved already at T2, IADL starts to improve only at T4. Complete autonomy is restored for both scales at T7.

Regarding radiological vasospasm effects on cognition at T2, the T-tests showed a significant impact on several tests (note: no statistical differences emerged at T1 from patients with and without vasospasm): in the memory domain, patients with vasospasm had a significantly worse performance at the Corsi test (*p* = 0.024, t_(56)_ = 2.32, mean score 3.34 ± 1.02) and the figure recall test (*p* = 0.033, t_(56)_ = 2.19, mean score 6.82 ± 5.85), as compared with those who did not develop it (mean scores 3.88 ± 0.71 and 9.96 ± 5.08, respectively).

Patients without radiological vasospasm performed better also at the Token test (*p* = 0.025, t_(56)_ = 2.45, mean 31.03 ± 3.03) and at the phonemic fluency test (*p* = 0.027, t_(56)_ = 2.27, mean 24.16 ± 11.8) with respect to those with (mean 27.89 ± 6.34 and 17.25 ± 11.48, respectively), and, concerning the executive functions, at the clock drawing (*p* = 0.044, t_(56)_ = 2.09, mean 7.17 ± 3.35) and the stroop test, in both the time (*p* = 0.039, t_(53)_ = −2.17, mean 33.45 ± 19.12) and error scores (*p* = 0.032, t_(53)_ = −2.26, mean 1.98 ± 2.27). Patients with vasospasm obtained a mean score of 5.16 ± 3.96 at the clock drawing test, while a mean score of 75.89 ± 96.17 was found for the time score of the Stroop Test, showing a great difference between the two groups; their stroop error mean score was 5.27 ± 6.97, instead. Similarly, the Digit Span Backward was significantly more impaired (*p* = 0.046, t_(55)_ = 2.04, mean 2.45 ± 0.95) in patients with vasospasm, as compared to those without (mean 3.49 ± 0.94).

For the praxis domain, only the figure copy test showed significant (*p* = 0.030, t_(55)_ = 2.40) lower mean scores in the vasospasm group (23.50 ± 11.67 vs. 29.33 ± 6.75).

Finally, a significant impact of vasospasm was found also for the ADL (*p* = 0.003, U = 241.5) and IADL scales (*p* = 0.004, U = 241.5): Patients with vasospasm were significantly more impaired in both instrumental (mean 3.85 ± 2.1) and instrumental activities (mean 1.22 ± 1.08) than those who did not develop it (mean 5.29 ± 1.3, and 2.16 ± 1.21, respectively).

**Figure 1:**
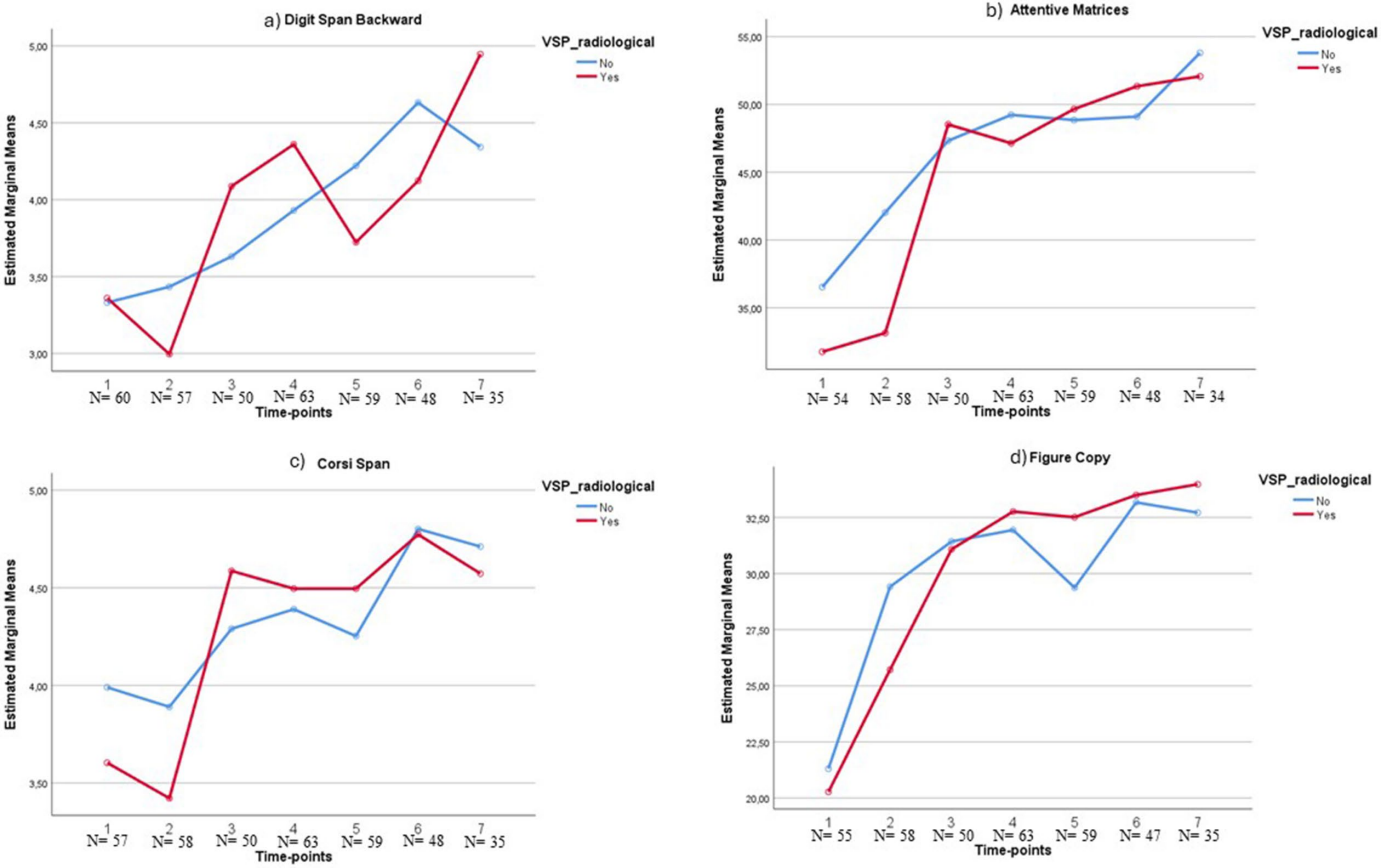
Example of recover trajectories of some of the tests administered. VSP_radiological: radiological vasospasm; *N* = number of patients tested at that time-point.

To further study the effects of vasospasm over time, a mixed repeated measures ANOVA with time and vasospasm as predictors was conducted, showing significant linear trends of improvement for time for all the tests administered, indicating a linear, gradual recovery over time for all the investigated functions. Only in the digit span backward a significant interaction effect (*p* = 0.023, *F*_(6)_ = 2.56; Mauchly’s test *p* = 0.095) between the two independent variables considered emerged. As shown in Figure 1a, whereas the test scores of patients without vasospasm gradually improve over time, a swinging trend emerges for patients with vasospasm: interestingly, in both of two groups, a significant difference (*p* = 0.004 and <0.001) between respective mean scores at T1 and T7 is reported. Moreover, in the vasospasm group, significant differences emerge between T2 and all the following time points (*p*-values ranging from <0.001 to a maximum of 0.038).

Noteworthy, patients who experienced vasospasm show evident improvement in most of the tests immediately at T3, often reaching at this time-point the same level as the other group, as shown in Figures 1b,c for example. Only a few tests show different qualitative trends: the RAVLT Immediate and Delayed scores show an anticipated recovery at T2, and similar scores at T6 (*p* > 0.05) and T7 (*p* > 0.05) in the two groups indicate a general recovery of verbal learning and long-term memory independently of vasospasm. Similarly, in the figure copy task, the improvement can be observed already at T2 also in the vasospasm group (Figure 1d).

## 4 Discussion

In this paper, we first aimed to deeply describe the neuropsychological functions over time in patients with aSAH, including levels of daily independence, focusing on the acute and sub-acute stages. In our opinion, this is of paramount importance, as aSAH incidence is higher during the 40–60 years of age, a range in which people are highly productive and have significant family and working responsibilities. Thus, a better understanding of cognitive and autonomy recoveries over time can be helpful for clinicians to explain and reassure patients and relatives concerning real expectations of impairments and quality of life after the aSAH and to tailor eventual rehabilitation programs.

The cognitive and functional deficits observed in this study underscore the profound and multifaceted impact of aSAH on brain function: the acute-phase impairments, particularly in memory, attention, and executive functions, are consistent with prior studies reporting widespread cortical and subcortical damage secondary to the initial hemorrhage, elevated intracranial pressure, and delayed ischemic events.

Controversial data are available concerning the most impactful factors on neuropsychological functions: previous (10, 19) and recent studies (20, 21) investigated a huge variety of variables, reporting no significant effects of the kind of intervention (clipping vs. coiling), aneurysm size, hypertension, neuroinflammation, intraoperative temperature, vasospasm, and the anterior or posterior circulation. In contrast to these results, a recent review (22) described a potential effect of posterior localization on memory deficits, together with the presence of acute hydrocephalus and the related necessity of cerebrospinal fluid drainage as a negative prognostic factor for cognition, similarly to another research (10) underlying the importance of proper fluid management in the acute phase to prevent neurological complications and consequent cognitive deterioration.

Considering bleeding localization, in almost half of our sample, the hemorrhage occurred on the anterior communicating artery, a localization that may be linked specifically to executive functions and behavioral deficits. Nevertheless, despite this anatomo-functional correlation, we found a wider variety of cognitive functions impaired besides frontal impairments. In this view, our results may sustain the hypothesis that cognitive deficits after aSAH may reflect the severity of the widespread and generalized brain tissue damage caused by bleeding and subsequent related events, rather than localized lesions as in other kinds of neurological conditions (for example, brain tumors or isolated ischemia) (10, 19–21).

With respect to recovery patterns across cognitive domains, different trajectories are depicted in our results, with visuospatial memory and executive functions showing slower recovery compared to attention and language, similarly to the results of a recent review (22). This heterogeneity suggests that specific neural circuits, such as the hippocampal and prefrontal networks, may be more susceptible to ischemic and inflammatory damage, requiring longer periods for neuroplastic reorganization.

An extensive review (13) reported a great number of cognitive deficits during the first 3 months after hemorrhage, with discordant results concerning long-term outcomes. In particular, the memory domain was reported to be most compromised, whose impairments also lasted after 18 months, especially for long-term verbal functions. Coherently, in our sample, the memory domain shows great impairments in the acute phase, markedly for verbal learning/long-term verbal memory, indicating an evident acute impact of bleeding over these functions; besides the eventual presence of vasospasm, in the sub-acute follow-up (T2), all tests improved their scores, except for the short-term visuospatial memory test (Corsi span), suggesting a slight effect of vasospasm over these functions, as confirmed by the other analyses. Gradual improvement emerges over time until T7, in which very low percentages of impairment were detected, in particular for the verbal short-term memory test, an element in line with previous reports (10). Only the long-term visuospatial memory task shows a higher prevalence of deficit at the 18-month follow-up, differently from another paper in which visual memory showed complete recovery after 1 year from aSAH (10), but similarly to a recent review (22) described visuo-spatial skills and memory domains as the most impaired at 1 year after aSAH.

Furthermore, the visual attention test, despite half of the sample scoring deficiently in the acute phase, shows significant improvements over time, with only one patient reporting impaired performance at 18 months.

In the acute phase, high prevalence of impairment is visible in the language domain, in particular for the phonemic fluency test; interestingly, already at T2, a general recovery can be found, and, at 18 months, almost all patients show normal performances in the language tests. A similar pattern emerges for the praxis functions, whereas the executive functions domain does not improve in the sub-acute phase, suggesting a greater impact of vasospasm on these kinds of cognitive abilities. Nevertheless, the executive domain also gradually improves over time, with very low percentages of impairment at the chronic stages. A greater variability of trends and impairments has been described (13, 22) for executive functions with respect to the other cognitive domains, which show more stable and gradual trends of improvement over time (12).

For daily autonomy, severe impairments are present in the acute phase, coherently with previous data (13), confirming the great impact of aSAH on functional status. Noteworthy, a complete recovery can be depicted at the chronic stages in our sample, with the ADL abilities recovering faster than the IADLs. Functional autonomy has been described as highly influenced by the severity of bleeding and particularly dependent on acute medical management and treatments (22).

In our sample, patients with vasospasm obtained significantly worse scores at T2 for long- and short-term visual memory, constructional apraxia, language comprehension, phonemic fluencies, verbal working memory, cognitive inhibition, and daily independence. Thus, vasospasm had a deep acute impact on the general cognitive functioning, and in particular on visuospatial abilities, as suggested by Corsi span, complex figure copy and recall, and the clock drawing tests. Nevertheless, while it exacerbates severe acute cognitive impairments, its long-term effects appear limited, in agreement with previous research, which failed to find direct effects of vasospasm on cognitive outcomes (10, 20, 22). The transient impact of vasospasm aligns with emerging evidence suggesting that global mechanisms such as neuroinflammation, rather than localized ischemia, may play a more critical role in determining chronic outcomes (23, 24). Nonetheless, the unstable recovery trends observed in working memory in patients with vasospasm raise questions about potential delayed or subtle effects of vasospasm on specific cognitive functions. Interestingly, these findings suggest that timely management of vasospasm may mitigate its acute impact, supporting the importance of interventions such as hemodynamic therapy, nimodipine administration, and close monitoring with transcranial Doppler. Moreover, the rapid recovery observed in some domains post-vasospasm highlights the resilience of certain cognitive networks, which may benefit from further targeted treatments and rehabilitation during this critical period.

These findings have significant implications for patient counselling and rehabilitation planning. For instance, clinicians can reassure patients and caregivers about the expected timeline of functional recovery while emphasizing the importance of continued cognitive and physical monitoring and therapy.

As reported above, our study indirectly supports the hypothesis that diffuse mechanisms such as neuroinflammation, diffuse axonal injury, and blood–brain barrier disruption may underlie the observed cognitive impairments and recovery patterns. Previous research has linked elevated levels of inflammatory biomarkers (e.g., interleukin-6 and C-reactive protein) with worse cognitive outcomes following aSAH (24, 25), but controversial results are available. Understanding these mechanisms through future dedicated studies could pave the way for further novel therapeutic approaches, such as anti-inflammatory treatments or neuroprotective agents, to enhance recovery.

In this view, incorporating regular neuropsychological assessments into standard care protocols could also help identify patients at risk of prolonged deficits and guide timely interventions.

In general, in our sample, the variable Time seems to be more efficient in predicting cognitive long-term outcomes than the presence of vasospasm, a finding coherent with other research (19): despite being a severe condition, patients who survive an aSAH and the first sequelae show a low prevalence of cognitive impairment and no impairments in daily autonomy at the chronic stages, a positive unobvious scenario already described in previous data (10). Even patients with vasospasm report slight long-term effects of this event, a reassuring element to take into account for clinicians. Additionally, our cognitive and functional outcomes underline the security and efficacy of the modern treatments available for this condition, particularly those provided during the acute phases.

Concerning limitations, a first methodological limit is represented by the reduction in sample size over time, which limited the statistical power of the longitudinal analyses. Future prospective studies might include a fixed number of patients for all the time points to study in order to obtain more powerful statistical data concerning long-term follow-ups.

Another limit is represented by the unequal distribution of some other clinical factors potentially influencing the results (for example, hydrocephalus and site of bleeding); this limitation prevented us from analyzing and exploring their effects and interactions, as no reliable analyses could be performed on them. Future studies with stratified recruitment or larger sample sizes could address this issue.

Moreover, in future research, the inclusion of advanced neuroimaging techniques, such as diffusion tensor imaging (DTI) or resting-state functional MRI, could provide deeper insights into the structural and functional changes associated with recovery.

## Data Availability

The raw data supporting the conclusions of this article will be made available by the authors, without undue reservation.
